# Electrochemistry Study of Bio-Based Composite Biopolymer Electrolyte—Starch/Cardol

**DOI:** 10.3390/polym15091994

**Published:** 2023-04-23

**Authors:** Alvaro A. Arrieta, Yamid Nuñez de la Rosa, Manuel Palencia

**Affiliations:** 1 Department of Biology and Chemistry, Universidad de Sucre (University of Sucre), Sincelejo 700001, Colombia; 2 Faculty of Engineering and Basic Sciences, Fundación Universitaria Los Libertadores, Bogotá 110231, Colombia; 3 Research Group in Science with Technological Applications (GI-CAT), Department of Chemistry, Faculty of Natural and Exact Sciences, University of Valle, Cali 760042, Colombia

**Keywords:** cassava starch, biopolymer, electroactivity, cardol, CNSL

## Abstract

The environmental problems generated by pollution due to polymers of petrochemical origin have led to the search for eco-friendly alternatives such as the development of biopolymers or bio-based polymers. The aim of this work was to evaluate the electrochemical behavior of a biopolymer composite made from cassava starch and cardol extracted from cashew nut shell liquid. The biopolymers were prepared using the thermochemical method, varying the synthesis pH and the cardol amounts. The biopolymers were synthesized in the form of films and characterized by cyclic voltamperometry and electrochemical impedance spectroscopy. The biopolymers showed a rich electroactivity, with three oxidation–reduction processes evidenced in the voltamperograms. On the other hand, the equivalent circuit corresponding to the impedance behavior of biopolymers integrated the processes of electron transfer resistance, electric double layer, redox reaction process, and resistance of the biopolymeric matrix. The results allowed us to conclude that the cardol content and the synthesis pH were factors that affect the electrochemical behavior of biopolymer composite films. Electrochemical processes in biopolymers were reversible and involved two-electron transfer and were diffusion-controlled processes.

## 1. Introduction

Polymer pollution is a growing environmental problem caused by the release of plastic and other synthetic materials into the environment. It is estimated that there are between 90 and 95 million metric tons of plastic waste in the world, and more than 60% ends up in landfills, oceans, and other natural habitats [1,2]. In addition to direct harm to wildlife, polymer pollution also has indirect effects on the environment, including the release of toxic chemicals into soil and water, which can harm human and animal health [2,3,4]. Due to the problems generated by polymers of petrochemical origin, in recent years polymers based on natural sources have attracted attention for being eco-friendly materials.

Biopolymers are a promising alternative to traditional petroleum-based polymers, since they are made from renewable and biodegradable raw materials. Biopolymers are made from materials such as starch, cellulose, and proteins, and have similar physical and mechanical properties to traditional polymers [5,6,7,8]. The use of biopolymers can help to reduce the carbon footprint associated with traditional polymers, since they are made from renewable resources and have lower energy consumption during production [9,10]. In addition, biopolymers have the advantage of being biodegradable, which means that they break down into natural substances, thus reducing the risk of environmental contamination. Furthermore, the use of agro-industrial waste in the production of biopolymers is a promising solution to reduce waste and promote sustainability in the agriculture industry. Agro-industrial waste materials, such as straw and fruit and vegetable peels, are abundant and can be converted into biopolymers with physical, chemical, and mechanical properties similar to those of traditional polymers [11,12,13,14]. In the literature, the use of coconut shells in the generation of activated carbon, the use of shrimp shells for the extraction of chitosan, and the use of cellulose extracted from the bark of wood to generate membranes, among others, have been reported [15,16,17]. The use of agro-industrial waste in the production of biopolymers can help to reduce waste and improve the economic viability of agriculture by creating new sources of income for farmers and promoting rural development.

Polymer electrolytes, also known as solid-state electrolytes, are attracting increasing attention due to their potential to replace traditional liquid electrolytes in batteries and other energy storage devices. Polymer electrolytes consist of a polymer matrix with an ionic salt, which conducts ions through the material and allows the flow of electrical charge. Polymer electrolytes have several advantages over liquid electrolytes, including greater safety, less leakage, and greater flexibility, among others. Biopolymer electrolytes, also known as biodegradable polymer electrolytes, are made from natural resources, such as starch, cellulose, and chitosan, among others [16]. The use of biopolymers in electrolytes offers several advantages, including greater sustainability, biocompatibility, and biodegradability compared to traditional petroleum-based polymer electrolytes. Biopolymer electrolytes made from starch show promising results in terms of conductivity and stability, making them suitable for use in energy storage devices [18].

Starch, a natural polysaccharide, has been widely studied for its potential as a feedstock in the production of biopolymers. Starch is abundant, renewable, and has a low environmental impact, making it an attractive alternative to traditional polymers. The production of biopolymers from starch implies the modification of its molecular structure through chemical or physical processes, resulting in the formation of new biopolymers with improved properties and applications [19,20]. Another substance that in recent years has been attracting interest for the production of biopolymers is cardol, due to its antioxidant, antifungal, and bactericidal properties and good chemical reactivity [21,22,23]. Cardol extracted from cashew nut shell liquid (CNSL) has been explored as a feedstock in the production of biopolymers. CNSL is a by-product of the cashew industry and is abundant and readily available in many regions. Cardol-based biopolymers exhibit good thermal stability and mechanical properties, making them suitable for applications in the packaging, agriculture, and biomedical industries [21,24,25].

Starch has been used in the elaboration of biopolymers, biopolymer electrolytes, and combined with other substances in composite biopolymers to improve its properties and expand its technological potential [26,27,28]. However, no references were found in the literature on composite biopolymers made from starch and cardol. Therefore, the aim of this work was the develop a bio-based composite polymeric electrolyte from starch/cardol and study its electrochemical properties by cyclic voltamperometry and electrochemical impedance spectroscopy to explore its potential as a composite biopolymer electrolyte.

## 2. Materials and Methods

### 2.1. Reagents

Reagents used were: methanol (CH_3_OH; Merck, 99.8%), glycerol (C_3_H_8_O_3_; Merck, 99.5%), calcium hydroxide (Ca(OH)_2_; Merck, 99.9%), glutaraldehyde (C_5_H_8_O_2_; Aldrich, 70.0%), polyethylene glycol 400 (C_2n_H_4n_ + 2O_n+1,n_; Merck, 98.5%), hydroxide sodium (NaCl; Merck, 99.9%), citric acid (C₆H₈O₇; Aldrich, 99.5%), ammonia (NH_3_; Merck, 99.8%), lithium perchlorate (LiClO_4_; Aldrich, 99.9%), ethyl acetate (C_4_H_8_O_2_; Merck, 99.8%), and anhydrous sodium sulfate (Na_2_SO_4_; Merck, 99.0%). Solutions were made and reactions were carried out with ultrapure water.

### 2.2. Extraction of Cardol and Cassava Starch

Cardol synthesis was performed following the method reported by Paramashivappa from CNSL extracted in the laboratory [29]. For the extraction of CNSL, cashew nut shells were crushed to a size of 2 mm and processed in a presser mill pressing machine at room temperature and the resulting oil was collected and used to carry out the synthesis of cardol. Two hundred grams of CNSL was dissolved in 1.2 L with constant stirring and 100 g of calcium hydroxide was added little by little. The solution was heated to 50 °C with constant stirring for 4 h. The precipitate was extracted and the supernatant was concentrated under low pressure until reaching 400 mL. Four hundred milliliters of ammonia (25%) were added to the methanolic solution and stirred for 15 min. Cardol was extracted by adding 400 mL of hexane/ethyl acetate (80:20). The organic layer was washed with 200 mL of hydrochloric acid (5%) and 200 mL of ultrapure water. The organic solution was dried with anhydrous sodium sulfate and concentrated to obtain pure cardol.

Cassava starch was extracted by the wet method, which involved crushing 500 g of peeled tubers with 100 mL of water in an industrial processor. To the mass obtained, 1 L of water was added and the mixture was homogenized. The mixture was filtered through a muslin cloth and the filtrate was left to rest for 24 h. The precipitate was dried in an oven for 48 h at 40 °C and then macerated and sieved, obtaining starch in the form of a bright white powder.

The synthesized cardol and cassava starch were identified by infrared spectroscopy and compared with commercial standards. In addition, the official methods of the International Association of Analytical Chemists were used for the verification of cassava starch purity and HPLC for cardol.

### 2.3. Biopolymer Composite Film Synthesis Process

The synthesis of the biopolymer composite was carried out by the thermochemical method. For this, 3.0 g of cassava starch was dispersed with stirring in 100 mL of water, of which the pH was adjusted. The pH of the water used to prepare the dispersions was adjusted to three values: 3.0, 7.0, and 11.0, and for this, 0.1 mol L^−1^ solutions of NaOH or HCl were used as required for the adjustment. The dispersion was heated to 75 °C with constant stirring at 1500 r.p.m. for 15 min. Subsequently, the solution was allowed to cool to room temperature to add lithium perchlorate (1.5 g) and plasticizers: glycerol (2.0 g), glutaraldehyde (1.5 g), and polyethylene glycol (2.0 g). In addition, cardol was added at this stage, and biopolymers were prepared with 2.0 g and 1.0 g at the three pH values and films without cardol (0.0 g) were also prepared. The solutions were heated to 70 °C using constant stirring for 15 min. After the heating process, the solutions were poured into Teflon Petri dishes, which were placed in an oven for 48 h at a temperature of 70 °C. The films formed were left undisturbed for 24 h at room temperature [30,31]. Each of the films was prepared in triplicate to report the average value of the measurements carried out.

### 2.4. Electrochemical Measurements for Biopolymer Composite Characterization

The biopolymers were analyzed at room temperature. The electrochemical behavior was studied by cyclic voltamperometry and electrochemical impedance spectroscopy. In both techniques, a dry cell consisting of two 1 cm^2^ (1 cm × 1 cm) stainless steel sheets adhered to two separate acrylic sheets was used, between which the 1 cm × 1 cm biopolymer samples were sandwiched. A dry cell was used to prevent the electrochemical behavior of the films from being affected by the solutions present in cells with commonly used liquid electrolytes. In this way, the observed response corresponded to the electrochemical activity of the polymeric electrolyte under study.

A configuration of four points (four electrodes) was used, in which two electrode terminals were connected simultaneously to each plate, including a counter electrode and reference electrode in one of the sheets and working electrode and sensor electrode in the other. The open circuit potential (0.01 V) was used as the reference potential. The measurements were performed with a Gamry 1010E galvanostat/potentiostat, controlled by the Gamry Framework software. Cyclic voltamperometry was performed in a potential range of −2.0 V to 2.0 V with an initial potential of 0.0 V and scan rates of 10 to 1000 mV s^−1^ were used. Electrochemical impedance spectroscopy was performed with an AC voltage of 10 mV rms and frequency range of 2 MHz to 10 mHz.

## 3. Results

**Figure 1:**
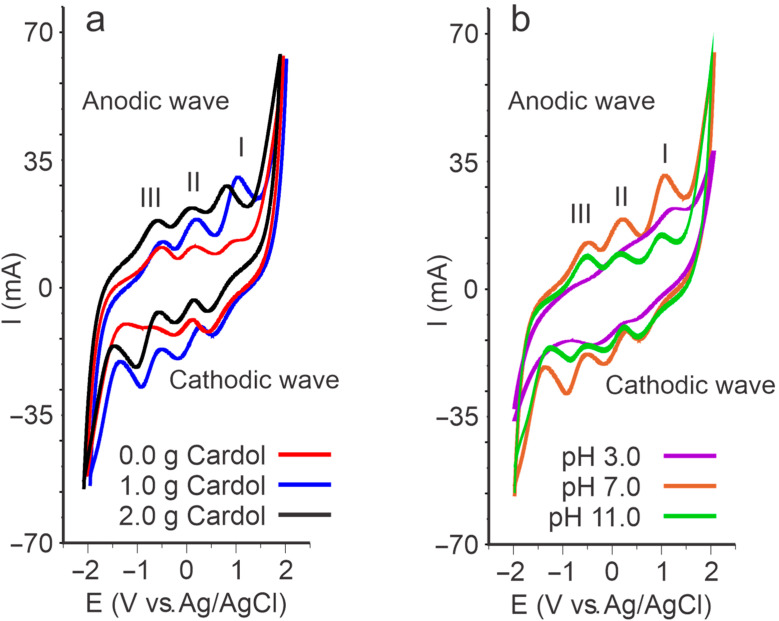
Cyclic voltamperometry of biopolymer films (**a**) synthesized at pH 7.0 with different concentrations of cardol and (**b**) synthesized with 1.0 g of cardol at different pH values.

The biopolymer films were easily detached from the Petri dishes and 1 cm^2^ samples were extracted for electrochemical measurements. Figure 1a shows, as a representative example of the behavior of the films made with different cardol concentrations, the voltamperograms of the biopolymers synthetized at pH 7.0. The behavior of the films made at different pH levels synthetized with 1.0 g of cardol is shown in Figure 1b. As can be seen in Figure 1a, the films containing cardol showed voltamperograms with well-defined redox processes and higher intensity than the films prepared without cardol.

In general, three redox couples were observed with three oxidation peaks in the anodic wave, paired with their corresponding reduction peak in the cathodic wave. The voltamperometric responses revealed the electroactive nature of the biopolymers.

The biopolymer synthesized without cardol (0.0 g) at pH 11.0 presented a voltamperogram with oxidation peaks at 1034.1 mV, 226.2 mV, and −489.9 mV, with their respective reduction peaks in the cathodic wave at 531.7 mV, −142.9 mV, and −949.1 mV. The signal of the biopolymer prepared with 1.0 g of cardol showed three oxidation peaks at 986.1 mV, 146.0 mV, and −512.1 mV, while the reduction peaks were shown at 504.3 mV, −222.1 mV, and −966.2 mV. On the other hand, the biopolymer with 2.0 g of cardol presented an anodic wave with peaks at 964.2 mV, 156.2 mV, and −520.1 mV and a reduction wave with peaks at 536.2 mV, −234.0 mV, and −972.5 mV. The voltamperometric signal of the biopolymer without cardol was less intense, presenting lower current values, which could indicate that the presence of cardol increases the conductivity in the films and improves the electroactivity. In addition, the presence of cardol can affect the oxidative and reductive potential, evidenced by the variation in the potentials of the oxidation and reduction peaks.

On the other hand, the voltamperograms of the films prepared with 1.0 g of cardol at different pH levels showed that the synthesis pH was an important factor in the electrochemical performance of the biopolymer. The biopolymer prepared at pH 3.0 did not show the three redox processes observed with the biopolymers synthesized at pH 7.0 and 11.0. The voltamperometric signal of the biopolymer synthesized at pH 3.0 presented a well-defined oxidation peak in the anodic wave at 1113.0 mV and a poorly resolved one at 48.2 mV, while the one in the cathodic wave a peak was defined at 324.4 mV and there was a low intensity one at −388.9 mV. The voltamperogram of the film synthesized at pH 7.0 showed three oxidation peaks at 1026.1 mV, 154.1 mV, and −568.0 mV and three reduction peaks at 520.2 mV, −226.0 mV, and −964.1 mV. The biopolymer synthesized at pH 11.0 presented a voltamperogram with three well-defined oxidation peaks at 986.1 mV, 146.0 mV, and −512.1 mV and their respective reduction pairs at 504.3 mV, −222.1 mV, and −966.2 mV. These variations in the electrochemical behavior of biopolymers may be due to differences in the polymeric structure and the crystallinity generated by the pH of synthesis [32,33].

The voltamperometric signals show that the electrochemical behavior of biopolymers was affected by the cardol content and the pH used in their synthesis process. Table 1 shows the anodic (Epa) and cathodic (Epc) peak potential values and the anodic (Ipa) and cathodic (Ipc) peak currents of the redox processes shown by the biopolymers prepared under different pH conditions and cardol concentrations.

In Table 1 it can be appreciated that, in general, the peak currents were slightly different without a clearly defined trend in the biopolymers with cardol. In the cardol-free starch biopolymer, a slight tendency to present a higher peak current at lower pH was observed. Therefore, the highest current values could be achieved at 3.0 pH and the lowest at 11.0 pH in cardol-free biopolymer. This was consistent with previously reported results, in which it was established that the synthesis pH in starch films affects the crystalline structure of the starch biopolymer, which directly influenced the displacement of charges and their conductivity [32].

Additionally, it was possible to appreciate that the peak currents were slightly higher in the biopolymers with cardol. This behavior may be due to the fact that the lower crystallinity of the starch biopolymer generated by the acidic pH (3.0) allowed a greater mobility of the charged species. In the case of the films with cardol, this behavior was altered by the presence of cardol, possibly because the cardol molecule was able to interact with the starch chains, making them less crystalline and less sensitive to pH effects than those that did not contain cardol.

On the other hand, the peak potentials also presented differences in terms of the pH and the concentration of cardol used. The biopolymers synthesized at lower pH presented lower values of peak potentials (Epa) in the oxidation processes and higher values in the reduction processes (Epc), which represent a lower oxidative capacity of the biopolymer.

**Figure 2:**
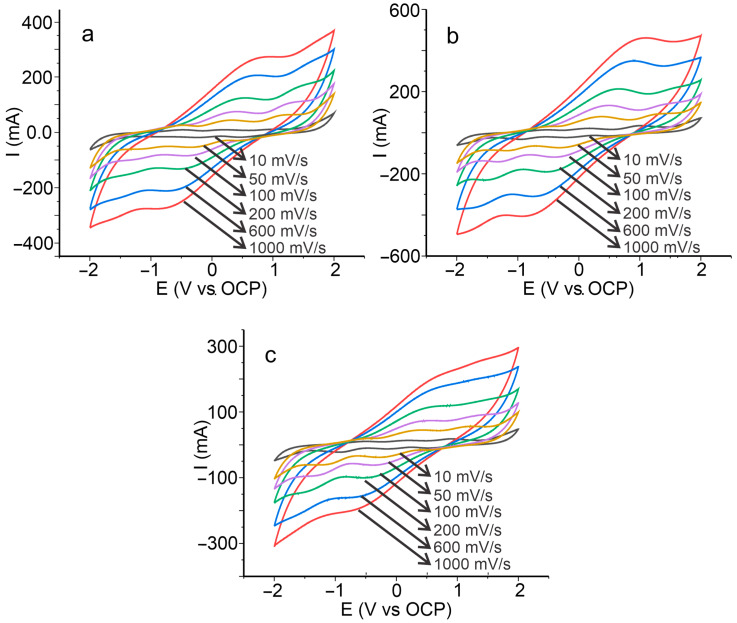
Cyclic voltamperometry of biopolymer films synthesized at pH 7.0 with (**a**) 0.0 g cardol (without cardol), (**b**) 1.0 g cardol, and (**c**) 2.0 g cardol, recorded with potential sweeps from 10 to 1000 mV s^−1^.

To understand the behavior and reversibility of biopolymer films, the effect of scan rate on electrochemical behavior was studied. Figure 2 shows the representative cyclic voltamperograms of the biopolymers produced at pH 7.0 with several cardol concentrations recorded at a scan rate from 10 to 1000 mV s^−1^.

It was observed that the currents of the redox processes increase with increasing scan rate. Furthermore, the oxidation peaks slightly tend to a positive direction and the reduction peaks to a negative direction as the scan rate increased. In addition, the peak heights (Ip) of the anode and cathode waves increase proportionally with the increase in scan rate with a fairly similar proportion.

**Figure 3:**
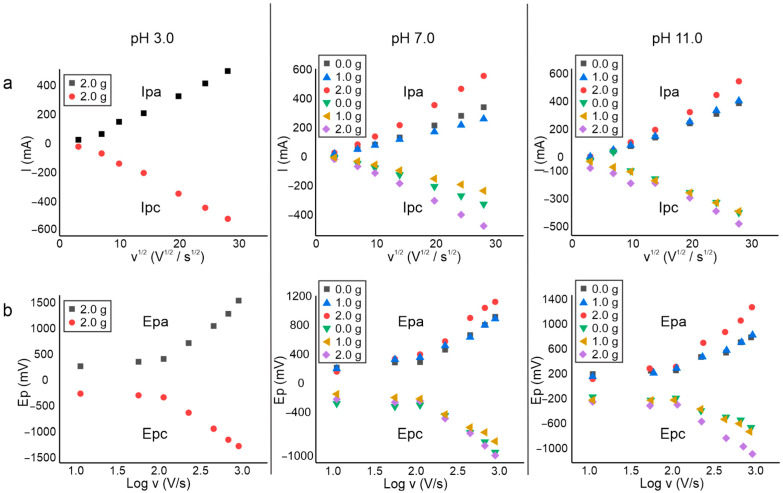
Correlation graphs of (**a**) peak currents (Ip) as a function of the square root of the scan rate (*v*^1/2^) and (**b**) peak potentials (Ep) as a function of the logarithm of the scan rate (log v).

The influence of the scan rate on the electrochemical behavior of the biopolymer was investigated by the establishment of the relationships between the peak (Ip) currents and peak potentials (Ep) with the scan rate. In Figure 3, the graphs of the relationship of the anodic peak current (Ipa) and the cathodic peak (Ipc) are presented with the square root of the scan rate (v^1/2^) (Figure 3a) and the relations of the peak potentials of anodic peaks (Epa) and the potential of cathodic peaks (Epc) with the scan rate logarithm (log v) are also presented (Figure 3b).

A linear relationship between the Ip was established with the square root of the scan rate; Ip (mA) = m (intersection) + Kp (slope) v^1/2^ (V^1/2^/s^1/2^). The correlation coefficients (R2) were in ranges of 0.971 to 0.998. In good agreement with previously reported results, the linear relationship suggests that electrochemical reactions were diffusion-controlled processes [34,35]. The slopes of the currents of anodic peaks (Ipa) and cathodic peaks (Ipc) were very alike in each electrochemical process, which indicates that the oxidation and reduction reactions occur at a similar speed of electron transference.

In the study of the relationship between Ep and logarithm of the scan rate (Figure 3b), the peak potentials increase in a linear relationship with log v. It has been established that this behavior is typical of heterogeneous electron transfer processes, since the processes change from reversible to quasi-irreversible at slow to fast scan rates. The relationship of Ep and log v was established by the regression equation as Ep (*mV*) = m (*intercept*) + Kp (*slope*) log v (*V*/*s*). The R2 values were found in a range of 0.942 to 0.998, based on the values determined for the correlation slopes Kp (*dEp*/*dlog* v) and taking into account what is established by the Laviron equation.

(1) $$Epc=E{}^{\circ}-\frac{2.3RT}{\alpha nF}log\;v$$

(2) $$Epa=E{}^{\circ}+\frac{2.3RT}{\left(1-\alpha \right)nF}log\;v$$

where R is the gas constant (8.314 J mol^−1^ K^−1^), T is temperature (K), α is the electron transfer coefficient, n is the number of electrons, and F is Faraday’s constant (96,493 C mol^−1^). From Equations (1) and (2), it was possible to calculate the charge transfer (α) and the number of electrons transferred (n). In all cases, the values of n and α were close to 2 and 0.5, respectively. These values could indicate that the electrochemical reaction involves the transfer of two electrons. The transfer of two electrons is an unlikely process, so this type of experimental behavior can be interpreted as the transfer (uptake or release) of one electron, followed by the transfer of a second electron or a chemical step.

Table 2 shows the values of the intersections and slopes obtained by plotting the peak currents Ip(a,c) as a function of the square root of the scan rate v^1/2^. It could be observed that in each biopolymer, regardless of the concentration or pH of synthesis, the values of the slopes were very similar for the anodic/cathode redox pairs (Ipa/Ipc), which could be due to the fact that the kinetics of the reaction oxidation and reduction were similar. Otherwise, the slopes of the films prepared with pH 7.0 were lower than those prepared at pH 11.0, indicating faster kinetics at basic pH. Additionally, biopolymers with a higher amount of cardol showed higher trend values, so the presence of cardol seems to facilitate the kinetics in biopolymers.

Table 3 shows the values of the intersections and slopes obtained with the peak potentials (Ep) as a function of the logarithm of the scan rate. It was possible to appreciate in all cases that there was symmetry in the slopes of the anodic and cathodic peaks, because their values were quite similar. Regarding the effects of pH, it was observed that at pH 11.0 the highest slope values were obtained, which could indicate that this pH favors the reversibility of electrochemical reactions in biopolymers synthesized under this condition. In addition, the amount of cardol used could also favor the reversibility of the processes due to the values of the slopes being higher than in the films prepared with 2.0 g of cardol.

**Figure 4:**
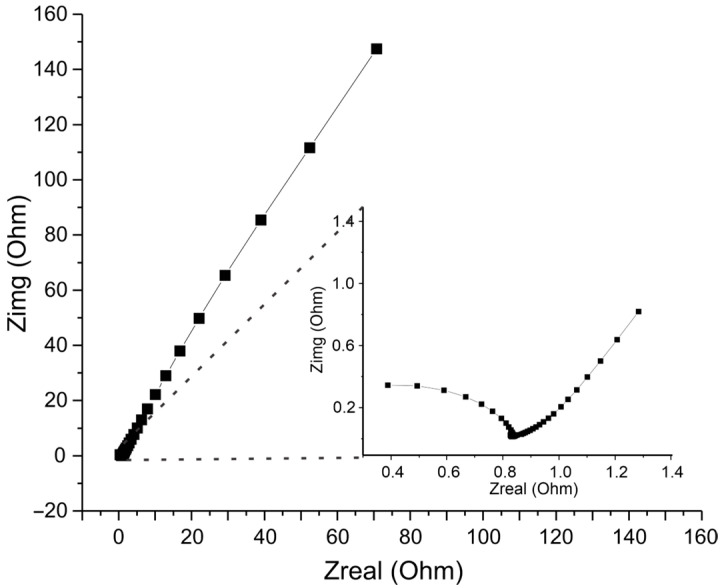
Nyquist graph of the biopolymer synthesized at pH 3.0 with 2.0 g of cardol. An enlargement of the first points is presented.

**Figure 5:**
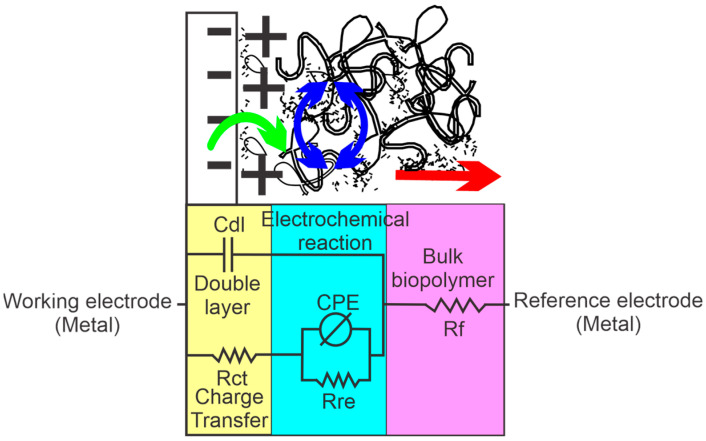
Scheme of the equivalent circuit corresponding to the impedance response of the biopolymers.

The electrochemical behavior of the films was also studied using electrochemical impedance spectroscopy. The impedance behavior of the films was similar in all cases. Figure 4 shows the Nyquist graph of the biopolymer film prepared at pH 7.0 with 2.0 g of cardol. In all cases, a semicircle (magnification in Figure 4) could be observed at high frequencies, followed by a slightly curved line at lower frequencies. This behavior was studied by establishing an equivalent circuit to establish a representation of the interface phenomenon where the exchange of electrons and electrochemical reactions occur. Figure 5 presents an equivalent circuit scheme constituted by a parallel resistance with a capacitor (Rct/Cdl), a parallel system composed of a constant phase element (CPE) with a resistance (Rre/CPE) connected in series with the Rct resistance, and, finally, a resistance (Rf) in series with the entire previous system. The equivalent electrical circuit can be summarized as [Rct(Rre/CPE)/Cdl]Rf.

In the upper part of Figure 5, a representation of the processes to which each element of the equivalent circuit corresponds is shown. The Rct resistance was due to the resistance of the charge transfer of the metallic electrode to the biopolymer, the Cdl capacitor generated by the charges of the electrode/biopolymer double layer, and a system in parallel of a resistance (Rre) and constant phase element, possibly due to the electrochemical reactions of oxidation/reduction that occurred in the biopolymers. In addition, the Rf resistance was due to the electrical resistance of the bulk of the biopolymer.

## 4. Conclusions

Composite biopolymers synthetized from cassava starch and cardol extracted from cashew nut shell liquid could be synthesized by an electrochemical method in the form of films using different pHs and cardol concentrations. The films were stable to mechanical traction and could be electrochemically characterized. The biopolymers were shown to be electroactive, presenting three well-defined reversible redox processes in composite biopolymers and less defined ones in cardol-free biopolymers. The speed of the electrochemical processes was controlled by diffusion and presented a reversible behavior at low scan rates and quasi-reversible behavior at scan rates greater than 100 mV s^−1^. The synthesis pH and the cardol concentration had an effect on the voltametric responses of the biopolymers, as the biopolymers prepared at pH 3.0 presented low electroactivity and lower conductivity than those prepared at pH 7.0 and 11.0. The cardol content favored the intensity of the voltamperometric signals and the diffusivity of charges in the biopolymers. It was established that the oxidation–reduction reactions implied the transfer of two electrons and that the equivalent circuit determined by electrochemical impedance spectroscopy was of the [Rct(Rre/CPE)/Cdl]Rf type and showed coherence with what was observed in the cyclic voltamperometry results.

## Figures and Tables

**Table 1:** Values of anodic and cathodic peak currents and potentials recorded in the biopolymer films by cyclic voltamperometry at scan rate of 10 mV s^−1^.

pH	Cardol (g)	Process I				Process II				Process III			
		Oxidation		Reduction		Oxidation		Reduction		Oxidation		Reduction	
		Ep	Ip	Ep	Ip	Ep	Ip	Ep	Ip	Ep	Ip	Ep	Ip
3	0.0	912.2	28.2	332.2	−24.7	666.0	21.5	−615.1	−23.7	--	--	--	--
	1.0	1113.0	19.8	324.4	−17.1	48.2	7.2	−388.9	−12.6	--	--	--	--
	2.0	854.2	19.9	586.1	−18.87	74.69	18.24	−246.1	−18.4	−590.1	10.2	−275.9	−18.6
7	0.0	1029.0	23.9	424.1	−15.5	208.2	22.5	−285.9	−22.4	−506.2	12.2	−931.5	−14.1
	1.0	1026.1	25.3	520.2	−15.8	154.1	20.95	−226.0	−23.2	−568.0	13.1	−964.1	−32.1
	2.0	918.2	25.9	528.0	−19.8	154.5	23.33	−155.8	−25.4	−509.9	13.9	−970.0	−32.3
11	0.0	1034.1	23.1	531.7	−8.2	226.2	23.78	−142.9	−25.0	−489.9	15.1	−949.1	−33.7
	1.0	986.1	24.1	504.3	−15.5	146.0	26.82	−222.1	−26.0	−512.1	18.5	−966.2	−34.3
	2.0	964.2	24.8	536.2	−19.9	156.2	26.84	−234.0	−28.8	−520.1	20.0	−972.5	−36.1

**Table 2:** Values of the intercept (m), slopes (Kp), and correlation coefficient (R2) of the peak current (Ip) as a function of the square root of the scan rate (v^1/2^).

pH	Cardol (g)	Ipa			Ipc		
		Intercept (m)	Slope (Kp)	R2	Intercept (m)	Slope (Kp)	R2
3	0.0	-	-	-	-	-	-
	1.0	-	-	-	-	-	-
	2.0	−51.02	16.02	0.985	58.29	−17.07	0.988
7	0.0	−21.56	9.57	0.998	25.19	−9.19	0.996
	1.0	−71.13	20.46	0.993	57.23	−18.68	0.993
	2.0	−44.83	13.01	0.992	37.52	−12.72	0.993
11	0.0	−52.65	15.55	0.993	92.31	−16.58	0.971
	1.0	−84.85	20.01	0.990	32.36	−19.41	0.974
	2.0	−51.05	16.17	0.994	42.99	−14.52	0.995

**Table 3:** Values of the intercept (m), slopes (Kp), and correlation coefficient (R2) of the peak potential (Ep) as a function of the logarithm of the scan rate (log v).

pH	Cardol (g)	Epa			Epc		
		Intercept (m)	Slope (Kp)	R2	Intercept (m)	Slope (Kp)	R2
3	0.0	-	-	-	-	-	-
	1.0	-	-	-	-	-	-
	2.0	−1518.7	329.7	0.982	1708.1	−379.8	0.998
7	0.0	−949.0	624.3	0.961	906.2	−582.0	0.980
	1.0	−1547.4	727.8	0.986	1441.5	−803.3	0.986
	2.0	−1266.3	744.7	0.995	1444.5	−819.8	0.992
11	0.0	−765.3	537.4	0.968	600.1	−414.2	0.963
	1.0	−835.0	512.3	0.983	726.4	−407.6	0.942
	2.0	−1280.6	386.9	0.966	1400.2	−347.8	0.997

## Data Availability

The data that support this work and its results are not available to be shared because they are under confidentiality agreements. Access to the data can be requested through an official document.
